# Polymorphism and Hirshfeld surface analysis of tetra­oxa[2]perfluoro­arene[2]triazine

**DOI:** 10.1107/S205698902500194X

**Published:** 2025-03-06

**Authors:** Tadashi Kawasaki, Akiko Hori

**Affiliations:** a Department of Applied Chemistry, Graduate School of Engineering & Science, 307 Fukasaku, Minuma-ku, Saitama-shi, Saitama 337-8570, Japan; University of Neuchâtel, Switzerland

**Keywords:** crystal structure, polymorphism, tetra­fluoro­phenyl­ene, triazine, Hirshfeld surface analysis

## Abstract

The title compound, tetra­oxa[2]perfluoro­arene[2]triazine (C_20_H_6_F_8_N_6_O_6_), composed of two tetra­fluoro­phenyl­ene and two triazine moieties connected by four oxygen atoms, was crystallized *via* slow evaporation of a di­chloro­methane solution, yielding two polymorphs: block- (**I**) and plate-shaped (**II**) crystals. Polymorph **I** (triclinic, *P*1, *V* = 516 Å^3^ at 173 K) was previously reported by Yang *et al.* [(2015. *Org. Lett.***15**, 4414–4417] whereas the newly identified polymorph **II** (triclinic, *P*1, *V* = 1085 Å^3^ at 100 K) shares the same space group but has a unit-cell volume twice as large, accommodating two symmetrically distinct mol­ecules, *Mol­ecule-1* and *Mol­ecule-2*, with a different mol­ecular arrangement. Since these crystals are expected to exhibit the characteristics of non-porous adaptive crystals, detailed analyses of inter­molecular inter­actions were conducted, revealing that C—F⋯π-hole inter­actions are more pronounced in **II** than in **I**. Hirshfeld surface analysis at 100 K revealed that the primary contributions to the crystal packing in polymorph **I** were F⋯F (17.1%), F⋯H/H⋯F (21.5%), C⋯H/H⋯C (6.3%), C⋯F/F⋯C (4.5%) and C⋯O/O⋯C (9.2%) inter­actions, whereas in polymorph **II**, these inter­actions were F⋯F (9.9% and 10.0%), F⋯H/H⋯F (20.9% and 26.5%), C⋯H/H⋯C (6.3% and 2.9%), C⋯F/F⋯C (8.5% and 10.0%) and C⋯O/O⋯C (4.9% and 4.6%) for *Mol­ecule-1* and *Mol­ecule-2*, respectively. Powder X-ray diffraction analysis indicates that polymorph **I** is the more stable crystalline form, predominantly obtained through rapid precipitation or by grinding the crystals.

## Abstract 2

Tetra­oxa[2]perfluoro­arene[2]triazine (C_20_H_6_F_8_N_6_O_6_), composed of two tetra­fluoro­phenyl­ene and two triazine moieties connected by four oxygen atoms, was crystallized *via* slow evaporation of a di­chloro­methane solution, yielding two polymorphs with block- and plate-shaped crystals.

## 1. Chemical context

Polymorphism, the ability of a compound to crystallize in multiple forms, is significant for its implications in mol­ecular recognition, separation processes, and the design of non-porous adaptive crystals (Jie *et al.*, 2018 ▸; Yan *et al.*, 2023 ▸). Among polymorphic systems, tetra­oxa[4]arene derivatives, which feature aromatic rings linked by heteroatoms, stand out due to their structural flexibility and diversity. Tetra­oxa[4]arene, 2,4,6,8-tetra­oxa-1,5(1,3),3,7(1,4)-tetra­benzena­cyclo­octa­phane (Zhou *et al.*, 2014 ▸), a cyclic mol­ecule comprising four phenyl­ene units bridged by oxygen atoms, is a notable example exhibiting polymorphism. Crystallization studies have shown that tetra­oxa[4]arene forms block-shaped crystals (monoclinic, *P*2_1_/*c*) and prismatic crystals (monoclinic, *P*2_1_/*n*), each displaying distinct mol­ecular arrangements and inter­molecular inter­actions (Ishida *et al.*, 2024 ▸). These features, particularly its ability to form non-porous adaptive crystals, highlight its potential as a solid-state host framework with mol­ecular recognition properties.

Accordingly, to investigate π-hole inter­actions in electron-deficient aromatic systems (Williams, 1993 ▸; Hori, 2012 ▸), we have focused on the title compound, a structurally related analogue of tetra­oxa[4]arene in which the phenyl­ene units are replaced by tetra­fluoro­phenyl­ene and triazine moieties (Yang *et al.*, 2013 ▸). This structural modification extends the chemical and structural diversity of tetra­oxa[4]arene derivatives, enabling exploration of the effects of fluorination and triazine substitution as π-hole systems (Shimizu *et al.*, 2009 ▸; Salonen *et al.*, 2011 ▸; Wang *et al.*, 2015 ▸; Politzer *et al.*, 2021 ▸) on the crystal packing and inter­molecular inter­actions. A previously reported polymorph of the title compound (triclinic, *P*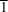) was noted for its potential adaptive crystal behavior. Inspired by the polymorphism observed in tetra­oxa[4]arene, we investigated the crystallization of the title compound and identified not only the previously reported block-shaped crystals, but also a novel plate-like form. This plate polymorph retains the same space group, but exhibits a unit-cell volume twice as large, accommodating a distinct mol­ecular arrangement. This study focuses on the crystallographic and structural characterization of the newly identified polymorph, aiming to elucidate its polymorphic behavior and inter­molecular inter­actions. Through this investigation, we seek to provide insights into the relationship between mol­ecular structure and polymorphism.
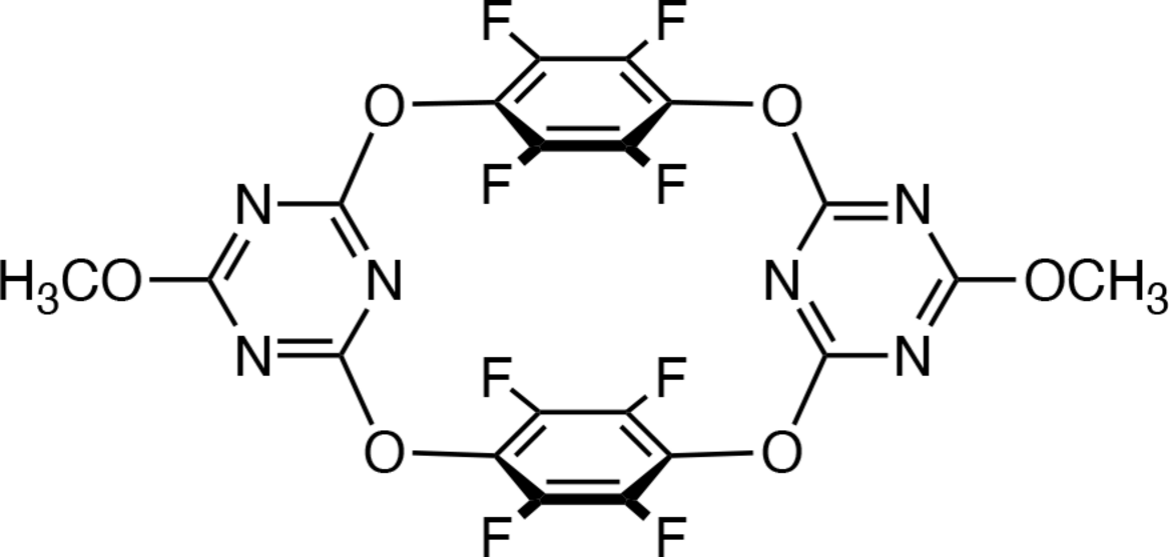


## 2. Structural commentary

The title compound was prepared following a reported method (Yang *et al.*, 2013 ▸) *via* a two-step coupling reaction between tetra­fluoro­hydro­quinone and 2,4-di­chloro-6-meth­oxy-1,3,5-triazine. After thorough purification, crystallization from a CH_2_Cl_2_ solution *via* slow evaporation yielded colorless block-shaped crystals (**I**) as the primary polymorph and plate-shaped crystals (**II**) as the minor polymorph. Repeated optimization experiments demonstrated that **II** could also be obtained as the primary polymorph under slow evaporation of the solvent, being isolated as the major polymorph.

**Figure 1:**
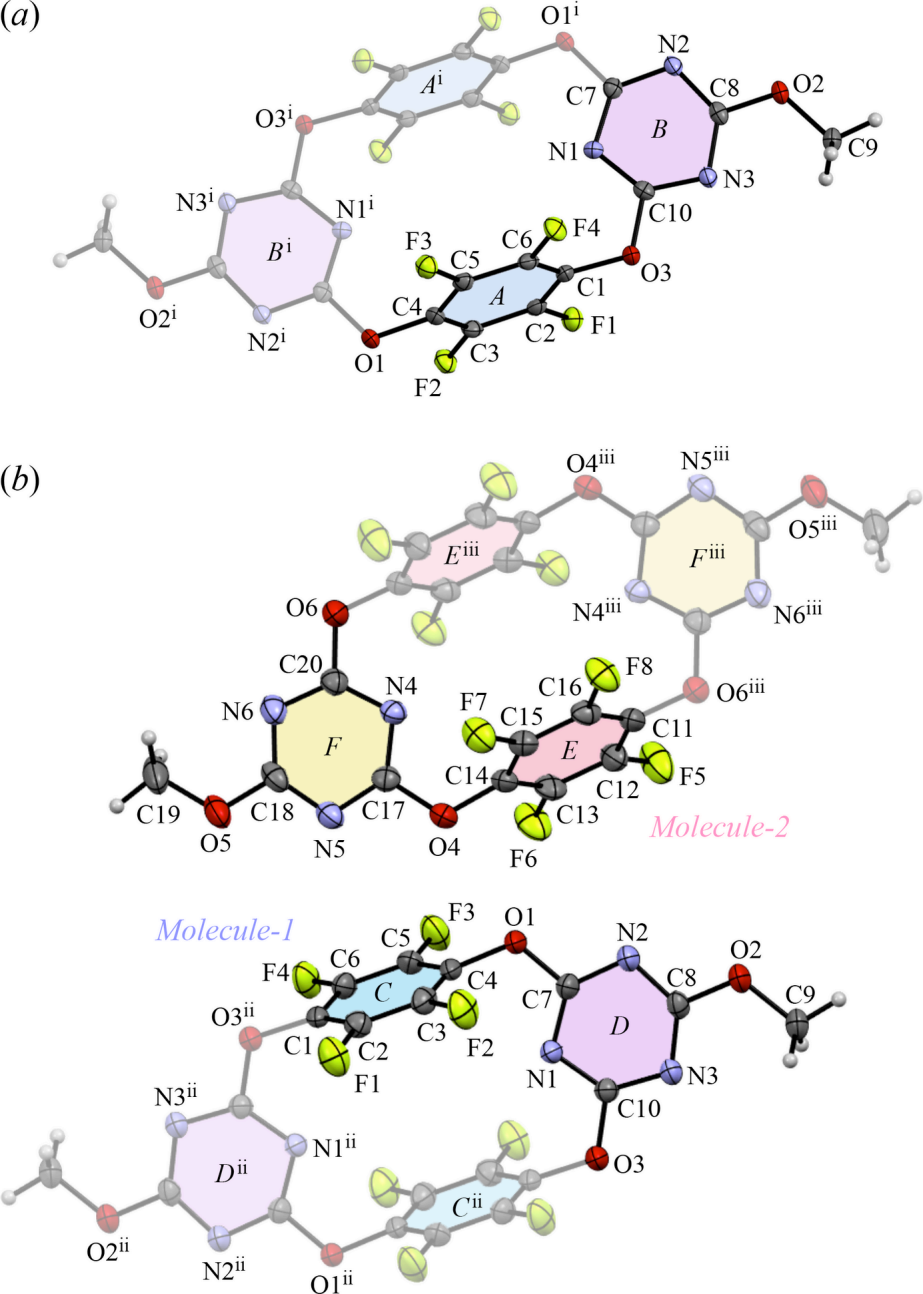
Mol­ecular structure of (*a*) **I** and (*b*) **II** at 100 K, showing the atom-labeling schemes. Displacement ellipsoids are drawn at the 50% probability level. Color scheme: C, gray; F, green; N, blue; O, red. Symmetry codes: (i) −*x* + 1, −*y* + 2, −*z* + 1; (ii) −*x*, −*y* + 1, −*z* + 1; (iii) −*x* + 1, −*y* + 1, −*z*.

Single-crystal structure analysis confirmed the polymorphic nature of the compound; the major block-shaped polymorph **I** (*P*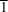) corresponds to the crystal structure previously reported by Wang *et al.* (2015 ▸) while the minor plate-like polymorph **II** also crystallizes in the same space group, but with a unit-cell dimension doubled along the *c*-axis. Although both polymorphs share the same crystal system and are centrosymmetric, they differ in their mol­ecular packing arrangements. To compare the inter­molecular inter­actions, a structural analysis was conducted for both crystals at 100 K. Additionally, the thermal stability of each crystal system was evaluated by examining their structural parameters at room temperature. The *ORTEP* representations of **I** and **II** at 100 K, along with their atom-labeling schemes, are shown in Fig. 1 ▸.

**Figure 2:**
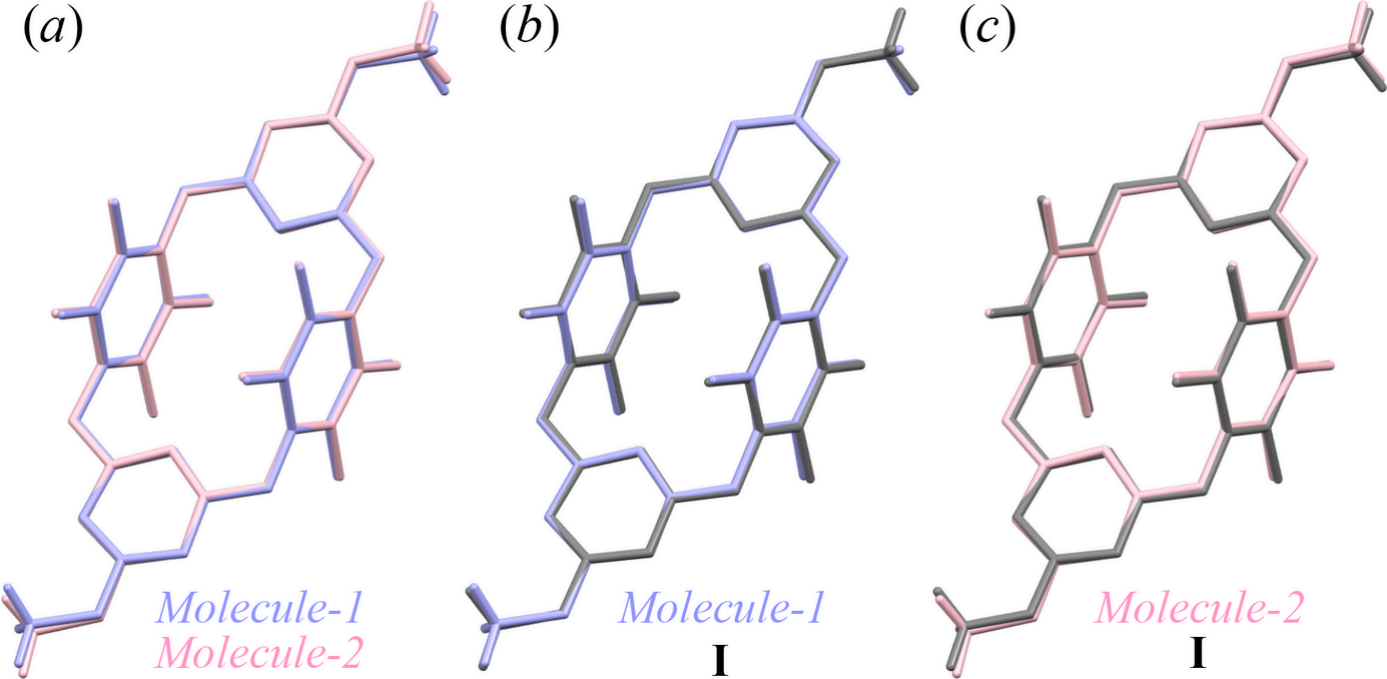
Structure overlays of the compound in the crystal of (*a*) *Mol­ecule-1* (in blue) and *Mol­ecule-2* (in pink) in **II**, (*b*) **I** (in gray) and *Mol­ecule-1*, and (*c*) **I** (in gray) and *Mol­ecule-2* at 100 K.

In polymorph **I**, the unit cell contains two half-mol­ecules as centrosymmetric units. The mol­ecule is a cyclic structure in which two tetra­fluoro­phenyl­ene rings and two triazine rings are alternately connected by oxygen atoms, with its inversion center located at the midpoint between them (Fig. 1 ▸*a*). The average C—O bond lengths and C—O—C bond angles for the oxygen atoms (O1 and O3) in the macrocyclic framework are 1.38 Å and 116°, respectively, indicating single bonds and localized π electrons. In polymorph **II**, the unit cell contains four half-mol­ecules, which are crystallographically independent and designated as *Mol­ecule-1* and *Mol­ecule-2* (Fig. 1 ▸*b*). The average C—O bond lengths and C—O—C bond angles of the framework are 1.37 Å and 116° for *Mol­ecule-1* (O1 and O3), and 1.38 Å and 116° for *Mol­ecule-2* (O4 and O6), respectively. Intra­molecular repulsion between fluorine atoms on opposing tetra­fluoro­phenyl­ene groups, such as F1 (Ring-*A*) and F3 (Ring-*A*^i^) [symmetry code: (i) −*x* + 1, −*y* + 2, −*z* + 1], and between fluorine and nitro­gen atoms on adjacent tetra­fluoro­phenyl­ene and triazine groups, such as F2 (Ring-*A*)⋯N1 (Ring-*B*)⋯F3 (Ring-*A*), contributes to the overall mol­ecular shape. The structural overlay of the cyclic frameworks, consisting of eighteen carbon, six nitro­gen, and four oxygen atoms, of *Mol­ecule-1* and *Mol­ecule-2* gives an r.m.s. deviation of 0.0564 Å (Fig. 2 ▸). Similarly, the r.m.s. deviations for the structural overlays of the cyclic frameworks in polymorph **I** compared with *Mol­ecule-1* and *Mol­ecule-2* in polymorph **II** are 0.052 and 0.0836 Å, respectively. As with tetra­oxa[4]arene, the mol­ecular structures are nearly identical, suggesting that the polymorphism observed originates not from differences in mol­ecular orientation, but rather from kinetically driven inter­molecular inter­actions during the crystallization process.

## 3. Supra­molecular features

**Figure 3:**
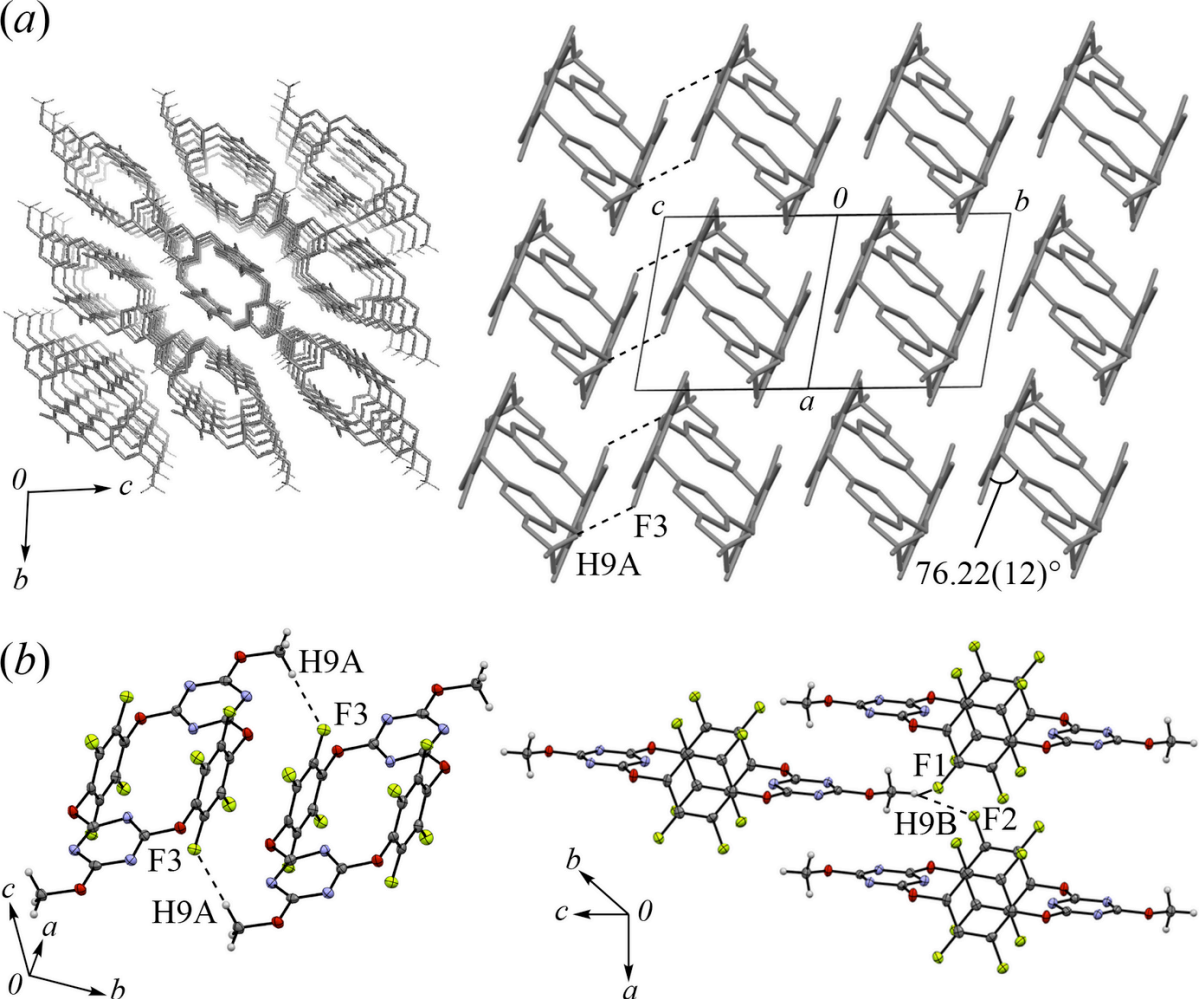
Partial packing views: (*a*) columnar arrangement along the *a*-axis (top view) and *bc*-direction (side view), (*b*) notable inter­molecular H⋯F inter­actions in **I**.

The packing structure of polymorph **I** and its key inter­molecular inter­actions are summarized in Fig. 3 ▸. The mol­ecules are aligned parallel to the (0
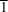
1) plane, forming a linear channel structure along the *a*-axis direction (Fig. 3 ▸*a*). Void analysis using *Mercury* (Macrae *et al.*, 2020 ▸) revealed that the radius of this channel is approximately 0.6 Å, indicating that it is not large enough for mol­ecular insertion. The dihedral angle between Ring-*A* and Ring-*B* was determined to be 76.22 (12)°, indicating that the opposing aromatic rings are positioned nearly parallel. Regarding inter­molecular inter­actions, H⋯F inter­actions were identified between H9*A*⋯F3, H9*B*⋯F1, and H9*B*⋯F2, with respective distances of 2.437, 2.630, and 2.601 Å, respectively (Fig. 3 ▸*b*). Other common inter­molecular inter­actions were scarcely detected in *PLATON* (Spek, 2020 ▸), while the short distances of C—H⋯π-hole, N⋯F, O⋯F, and F⋯F suggest that inter­molecular electrostatic inter­actions contribute to the crystal packing.

**Figure 4:**
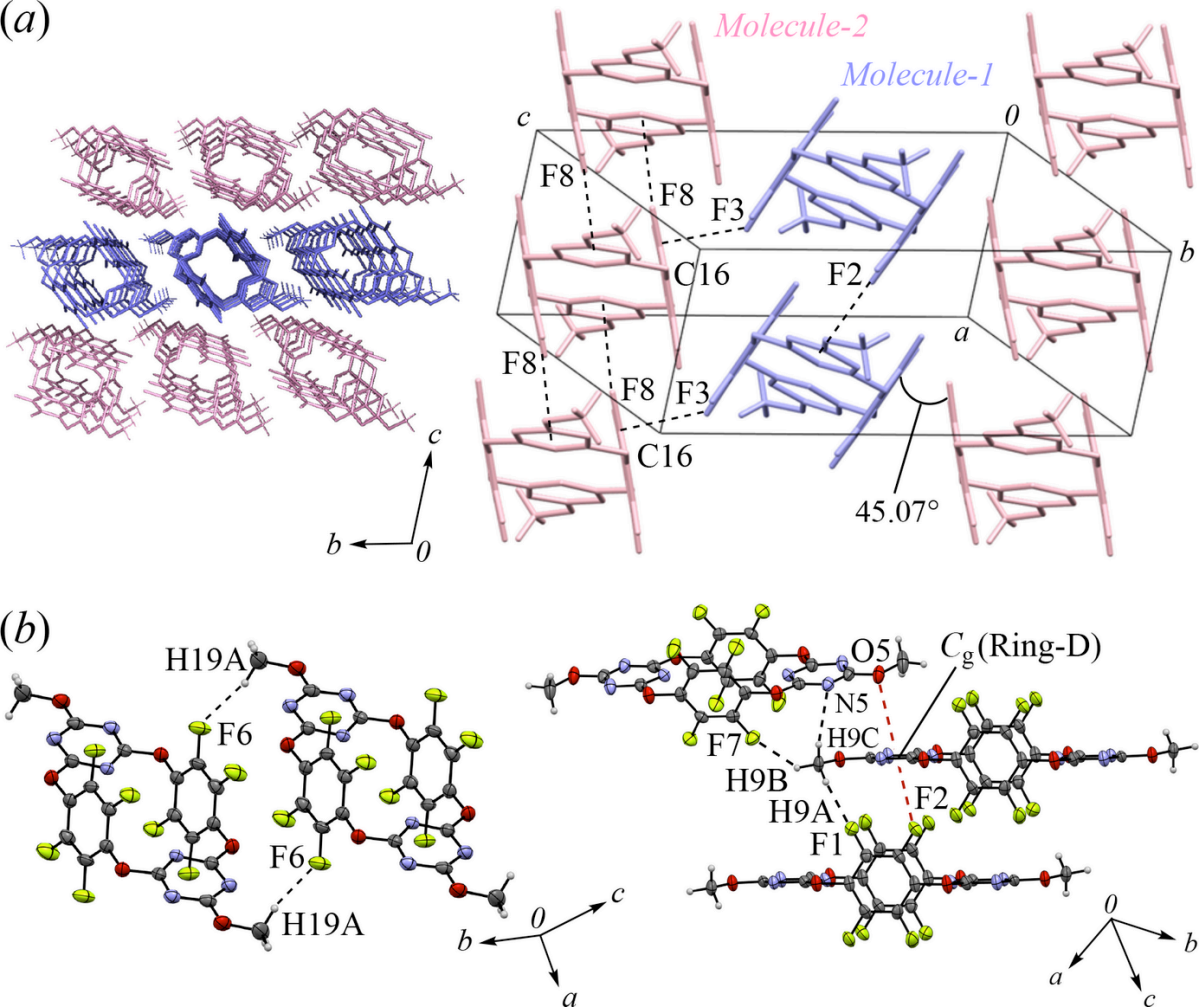
Partial packing views: (*a*) top and side views of the columnar arrangement along the *a*-axis, (*b*) notable inter­molecular H⋯F, H⋯N, C—F⋯π-hole, and O⋯π-hole inter­actions in **II**.

The packing structures and the corresponding inter­molecular inter­actions in **II** are shown in Fig. 4 ▸, where the two crystallographically independent mol­ecules, *Mol­ecule-1* and *Mol­ecule-2*, are identified in blue and pink, respectively, each being composed of two half-mol­ecules related by an inversion center [symmetry codes: (ii) −*x*, −*y* + 1, − *z* + 1; (iii) −*x* + 1, −*y* + 1, −*z*). The dihedral angles between the tetra­fluoro­phenyl­ene moieties of *Mol­ecule-1* and *Mol­ecule-2* are 45.07 (14)°, indicating a zigzag arrangement. Since both polymorphs **I** and **II** exhibit parallel π-hole planes within the mol­ecules, the compound is expected to form a co-crystal through π-hole⋯π inter­actions with aromatic guests *via* both the triazine and tetra­fluoro­phenyl­ene rings (Williams, 2017 ▸). The stabilization of these structures can be attributed to electrostatic repulsions observed in both polymorphs; several notable inter­molecular inter­actions were identified in **II**. The presence of C—F⋯π-hole inter­actions involving F2⋯*C*_g_ (Ring-*D*) and F8⋯*C*_g_ (Ring-*F*), as well as O⋯π-hole inter­actions observed between O5 and *C*_g_ (Ring-*D*), significantly contribute to the crystal packing: the inter­molecular distances are 3.136 (2) and 2.977 (2) Å for C—F⋯π-hole, and 3.585 Å for O⋯π-hole inter­actions. H⋯F inter­actions, such as H19*A*⋯F6, H9*A*⋯F1, and H9*B*⋯F7, and H⋯N inter­action, H9*C*⋯N5, were observed with corresponding distances of 2.412, 2.500, 2.633, and 2.739 Å. Similar to **I**, polymorph **II** also forms a linear channel structure along the *a*-axis direction. Void analysis revealed that the channel radius is approximately 0.6 Å, indicating that it is not large enough for mol­ecular insertion.

## 4. Hirshfeld surface analysis

**Figure 5:**
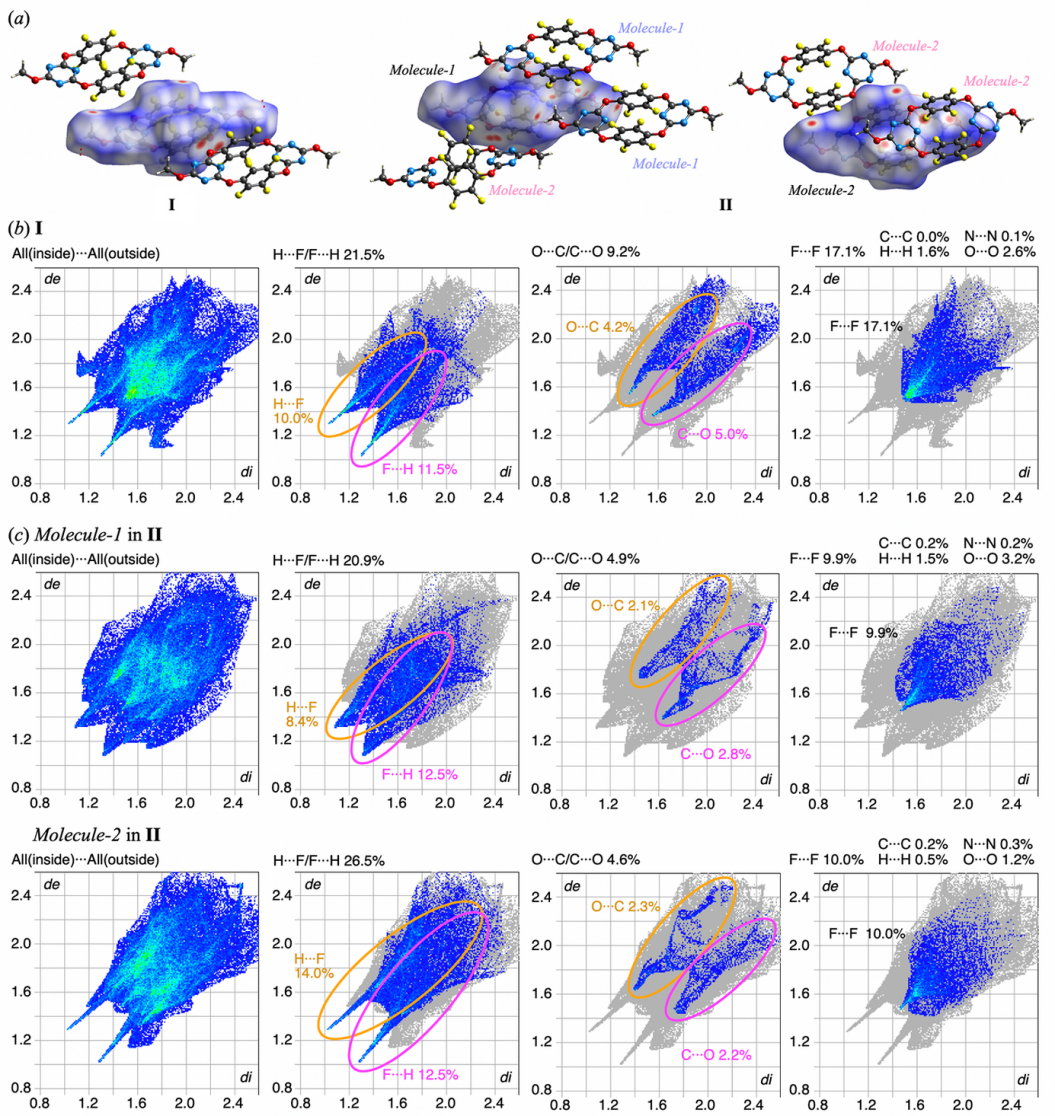
(*a*) Hirshfeld surface with *d*_norm_ of **I** and **II**. Fingerprint plots of (*b*) **I** and (*c*) **II**

To further investigate the strength of these inter­actions, Hirshfeld surface (HS) analysis was conducted (Hirshfeld, 1977 ▸; Spackman & Jayatilaka, 2009 ▸) using *Crystal Explorer 21.5* (Turner *et al.*, 2017 ▸). HS *d*_norm_ mapping of **I** and **II** (Fig. 5 ▸*a*) and fingerprint plots of **I** (Fig. 5 ▸*b*) confirmed that, in addition to the previously identified H⋯F/F⋯H inter­actions (21.5%), O⋯C/C⋯O inter­actions (9.2%) also play a significant role between the mol­ecules. The C⋯O contact distance was measured at 3.227 (3) Å, which is the same of the sum of the van der Waals radii (3.22 Å). Although *PLATON* (Spek, 2020 ▸) did not explicitly classify this as a notable inter­action, the adjacent carbon and oxygen atoms engage in C⋯O inter­actions, increasing the overall contribution of this inter­action to the crystal packing stability. No π-hole⋯π-hole stacking was observed due to the 0% contribution of C⋯C contacts.

For HS analysis of *Mol­ecule-1* and *Mol­ecule-2* in polymorph **II** (Fig. 5 ▸*c*), C—F⋯π-hole inter­actions were prominently observed, with a higher contribution of C⋯F/F⋯C (8.5% and 10.0%, respectively) in **II** compared to **I** (4.5%). Fingerprint plots were analyzed to focus on C⋯F/F⋯C and N⋯F/F⋯N inter­actions between fluorine atoms of the inner and the outer mol­ecules. This analysis was motivated by two key considerations: (i) the π-hole of the triazine ring consists of carbon and nitro­gen atoms, and (ii) C⋯F and N⋯F inter­actions that do not involve the π-hole contribution were rarely observed. The results showed that both *Mol­ecule-1* and *Mol­ecule-2* exhibited a relatively high contribution of C⋯F and N⋯F inter­actions, further supporting the dominance of C—F⋯π-hole inter­actions in **II**. Additionally, the H⋯F/F⋯H inter­actions, which were prominently observed in **I**, exhibited a greater contribution in **II**. However, the contribution of C⋯O/O⋯C inter­actions decreased. HS mapping revealed an increase in the number of strong inter­molecular inter­actions in **II**, suggesting that its lower symmetry arrangement resulted in a greater variety of inter­molecular inter­actions, leading to a more dispersed distribution of inter­action strengths.

## 5. Crystal growth and pXRD studies

**Figure 6:**
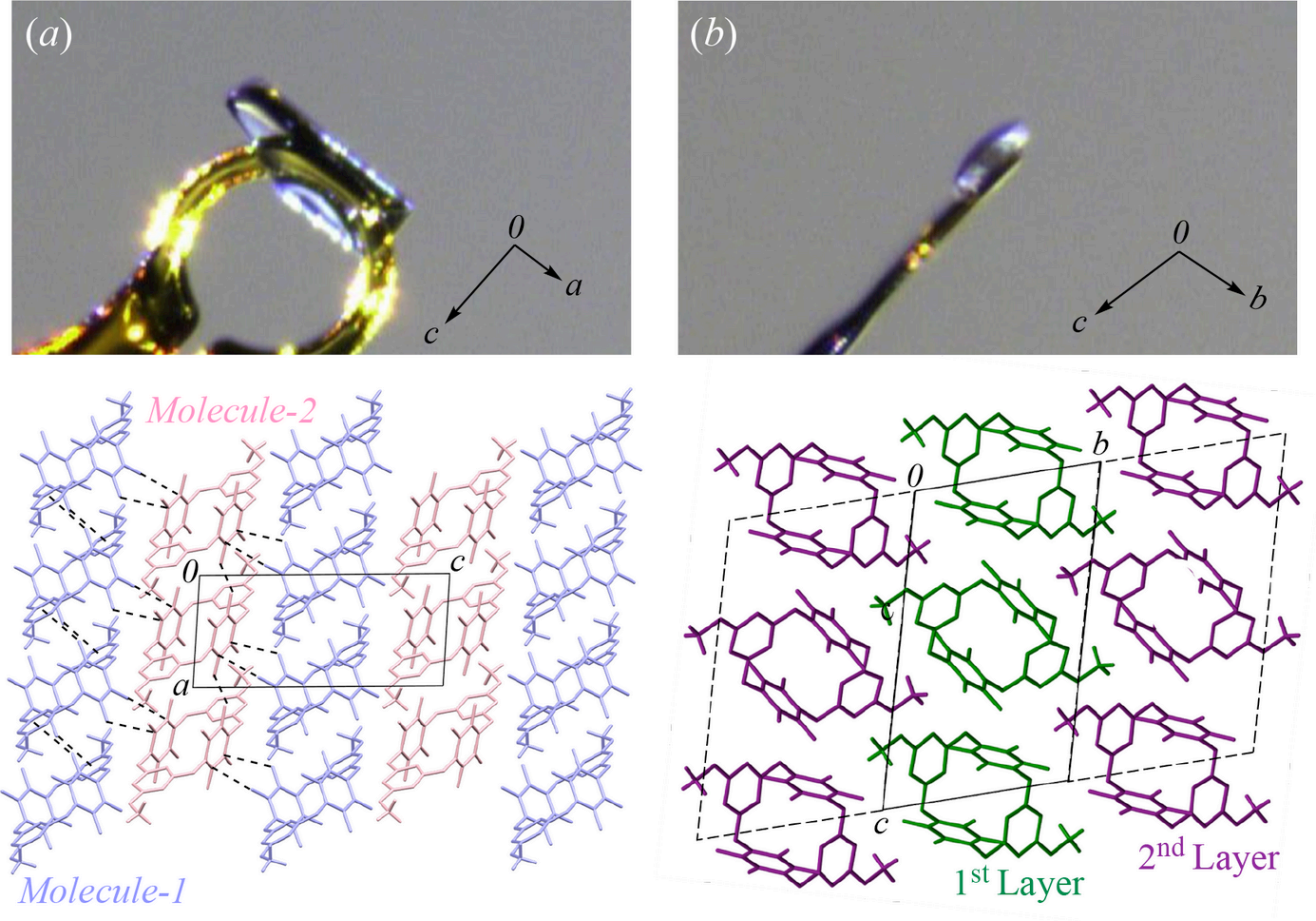
The crystal dimensions of polymorph **II**: views along (*a*) the *b*-axis and (*b*) the *a*-axis with the corresponding mol­ecular arrangements.

To investigate the driving force behind the plate-like crystal growth in this structure, growth packing plane analyses were performed. As shown in Fig. 6 ▸*a*, the crystal grows along both the *a*- and *c*-axes. Along the *a*-axis, *Mol­ecule-1* units are connected through C—F⋯π-hole inter­actions, while along the *c*-axis, *Mol­ecule-1* and *Mol­ecule-2* alternate, linked by the same type of inter­action. In contrast, Fig. 6 ▸*b* illustrates that the *b*-axis is the smallest dimension of the plate, the unit-cell layers extend without significant inter­molecular inter­actions. The formation of a distinct crystal system from polymorph **I** is attributed to the slow crystallization process, which enhances the dominance of C—F⋯π-hole inter­actions. At the same time, this process results in the emergence of growth-restricted planes, where these inter­actions are less effective. Consequently, the *b*-axis remains the direction of limited growth, leading to the formation of thin, plate-like crystals.

**Figure 7:**
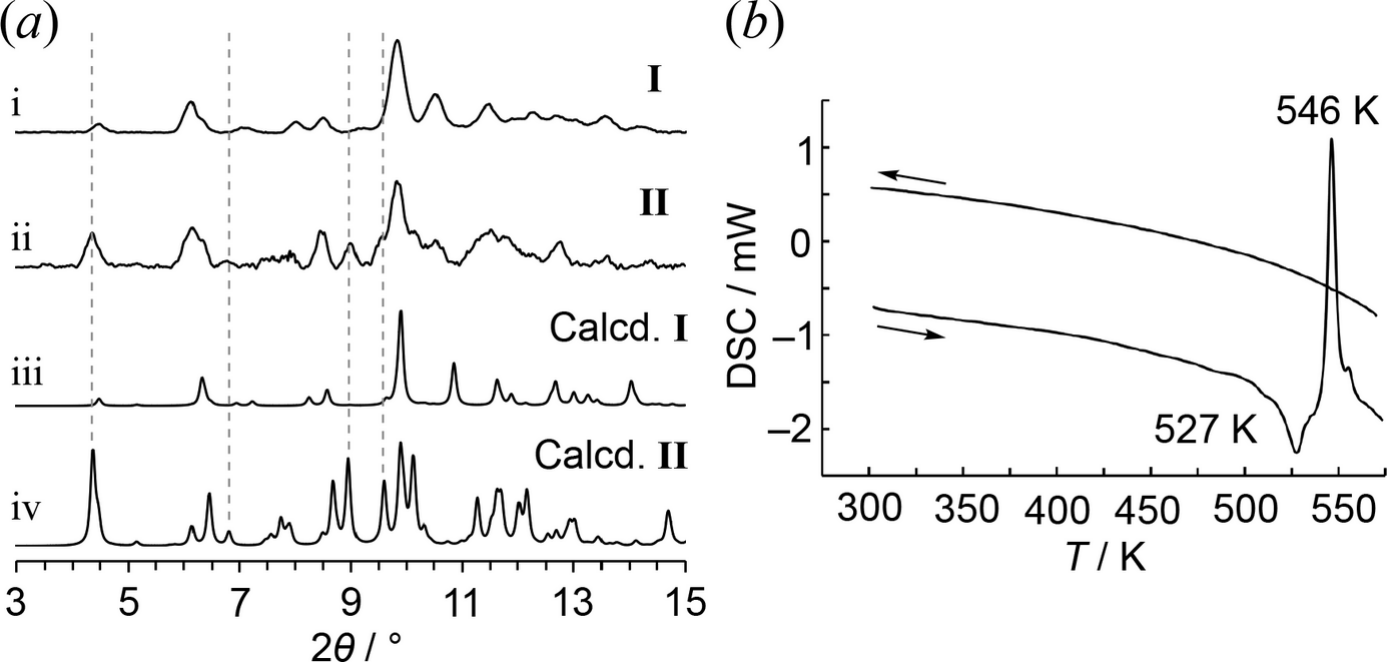
(*a*) pXRD patterns after grinding of (i) **I** and (ii) **II**; simulation patterns of (iii) **I** and (iv) **II** from scXRD, and (*b*) DSC of the title compound, scan rate: 10°C min^−1^.

To verify whether polymorphs **I** and **II** undergo other crystalline phase transitions, pXRD measurements (similar to scXRD) were performed (Fig. 7 ▸*a*). The powder pattern of the prepared sample (pattern-i) closely matched the pXRD simulation derived from the single-crystal structure of **I**, with no detectable pattern corresponding to **II**. The observed broadening and peak shifts were attributed to measurements at room temperature and the use of the glass capillary method. During recrystallization, it was found that rapid precipitation favored the formation of the more densely packed crystal **I** (*D_c_* = 1.875 g cm^−3^) compared to **II** (*D_c_* = 1.770 g cm^−3^). In contrast, when the powder was dissolved in CH_2_Cl_2_ and allowed to concentrate as slowly as possible, the majority of the resulting crystals were identified as **II**. The pXRD measurement of this system produced a mixed pattern-ii, showing both **I** and **II** simulations. The reproducibility of this result suggests that the crystals underwent a phase transition upon grinding. A structurally similar tetra­oxa[4]arene has been reported to undergo a phase transition at around 200 K for one of its polymorphic forms (Ishida, *et al.*, 2024 ▸). Therefore, the structure of **II** measured at 100 K was remeasured at r.t., but no significant changes in lattice parameters were observed. Similarly, no significant changes in lattice parameters were detected in **I** at both 100 K and r.t., as well as in the previously reported structure measured at 173 K. To further investigate, differential scanning calorimetry (DSC) was performed on the powdered sample (Fig. 7 ▸*b*), revealing an endothermic reaction around 527 K, suggesting melting. Additionally, an exothermic reaction was observed around 546 K; however, no peaks appeared during the cooling process, indicating that the compound had decomposed. Based on these findings, it was concluded that the compound does not undergo crystalline phase transitions upon temperature variation, and that the block-shaped polymorph **I** is generally the predominant form.

In summary, this study investigated the polymorphic behavior of the π-hole arene compound and its structural characteristics. Single-crystal analysis confirmed two polymorphs (**I** and **II**), both in the triclinic *P*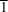 space group but with different unit-cell dimensions. While polymorph **I** corresponds to a previously reported structure, polymorph **II** has a doubled unit cell and two independent mol­ecules (*Mol­ecule-1* and *Mol­ecule-2*). C—F⋯π-hole inter­actions were found to be the dominant stabilizing force in both polymorphs. In **I**, these inter­actions contribute to a linear channel structure along the *a*-axis direction, with H⋯F inter­actions also playing a role. In **II**, C—F⋯π-hole inter­actions primarily link *Mol­ecule-1* and *Mol­ecule-2* along the *c*-axis, while H⋯F inter­actions show a higher contribution than in **I**. HS analysis further revealed C⋯F and N⋯F inter­actions, supporting the influence of triazine-based π-hole inter­actions. Growth-plane analysis indicated that slow crystallization enhances C—F⋯π-hole inter­actions, leading to plate-like crystal formation. These findings underscore the crucial role of π-hole inter­actions in crystal packing and polymorphism, providing insights into mol­ecular recognition and crystal engineering.

## 6. Refinement

Crystal data, data collection and structure refinement details are summarized in Table 1 ▸. H atoms were placed in geometrically idealized positions and refined as riding atoms with C—H = 0.95 Å and 0.98 Å for aromatic and aliphatic hydrogen, with *U*_iso_(H) = 1.2*U*_eq_(C).

## Supplementary Material

Crystal structure: contains datablock(s) global, I, II. DOI: 10.1107/S205698902500194X/tx2095sup1.cif

Structure factors: contains datablock(s) I. DOI: 10.1107/S205698902500194X/tx2095Isup2.hkl

Structure factors: contains datablock(s) II. DOI: 10.1107/S205698902500194X/tx2095IIsup3.hkl

CCDC references: 2427768, 2427767

Additional supporting information:  crystallographic information; 3D view; checkCIF report

## Figures and Tables

**Table 1:** Experimental details

	**I**	**II**
Crystal data		
Chemical formula	C_20_H_6_F_8_N_6_O_6_	C_20_H_6_F_8_N_6_O_6_
*M*_r_	578.31	578.31
Crystal system, space group	Triclinic, *P* 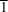	Triclinic, *P* 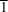
Temperature (K)	100	100
*a*, *b*, *c* (Å)	7.1461 (19), 8.660 (2), 9.280 (3)	7.1729 (10), 9.7282 (12), 16.307 (2)
α, β, γ (°)	90.742 (9), 101.274 (9), 113.887 (9)	104.420 (4), 91.293 (5), 99.274 (4)
*V* (Å^3^)	512.3 (2)	1085.3 (3)
*Z*	1	2
Radiation type	Mo *K*α	Mo *K*α
μ (mm^−1^)	0.19	0.18
Crystal size (mm)	0.18 × 0.12 × 0.06	0.25 × 0.19 × 0.09

Data collection		
Diffractometer	Bruker D8 Goniometer	Bruker D8 Goniometer
Absorption correction	Multi-scan (*SADABS*; Krause *et al.*, 2015 ▸)	Multi-scan (*SADABS*; Krause *et al*., 2015 ▸)
*T*_min_, *T*_max_	0.84, 0.99	0.74, 0.98
No. of measured, independent and observed [*I* > 2σ(*I*)] reflections	3886, 1723, 1324	10388, 3800, 2824
*R*_int_	0.030	0.053
(sin θ/λ)_max_ (Å^−1^)	0.595	0.595

Refinement		
*R*[*F*^2^ > 2σ(*F*^2^)], *wR*(*F*^2^), *S*	0.038, 0.092, 1.07	0.047, 0.130, 1.04
No. of reflections	1723	3800
No. of parameters	182	364
H-atom treatment	H-atom parameters constrained	H-atom parameters constrained
Δρ_max_, Δρ_min_ (e Å^−3^)	0.22, −0.28	0.26, −0.24

Computer programs: *APEX5* and *SAINT* (Bruker, 2019 ▸), *SHELXT2018/2* (Sheldrick, 2015*a* ▸), *SHELXL2019/1* (Sheldrick, 2015*b* ▸), *ShelXle* (Hübschle *et al.*, 2015 ▸) and *Mercury* (Macrae *et al.*, 2020 ▸).

## supplementary crystallographic information

### Tetraoxa[2]perfluoroarene[2]triazine (I) Crystal data

**Table:**

C_20_H_6_F_8_N_6_O_6_	*Z* = 1
*M**_r_* = 578.31	*F*(000) = 288
Triclinic, *P*1	*D*_x_ = 1.875 Mg m^−^^3^
*a* = 7.1461 (19) Å	Mo *K*α radiation, λ = 0.71073 Å
*b* = 8.660 (2) Å	Cell parameters from 1960 reflections
*c* = 9.280 (3) Å	θ = 3.2–26.3°
α = 90.742 (9)°	µ = 0.19 mm^−^^1^
β = 101.274 (9)°	*T* = 100 K
γ = 113.887 (9)°	Block, colourless
*V* = 512.3 (2) Å^3^	0.18 × 0.12 × 0.06 mm

### Tetraoxa[2]perfluoroarene[2]triazine (I) Data collection

**Table:**

Bruker D8 Goniometer diffractometer	1324 reflections with *I* > 2σ(*I*)
Detector resolution: 7.3910 pixels mm^-1^	*R*_int_ = 0.030
φ and ω scans	θ_max_ = 25.0°, θ_min_ = 3.2°
Absorption correction: multi-scan (SADABS; Krause et al., 2015)	*h* = −8→8
*T*_min_ = 0.84, *T*_max_ = 0.99	*k* = −9→10
3886 measured reflections	*l* = −11→10
1723 independent reflections	

### Tetraoxa[2]perfluoroarene[2]triazine (I) Refinement

**Table:**

Refinement on *F*^2^	0 restraints
Least-squares matrix: full	Hydrogen site location: inferred from neighbouring sites
*R*[*F*^2^ > 2σ(*F*^2^)] = 0.038	H-atom parameters constrained
*wR*(*F*^2^) = 0.092	*w* = 1/[σ^2^(*F*_o_^2^) + (0.0367*P*)^2^ + 0.2238*P*] where *P* = (*F*_o_^2^ + 2*F*_c_^2^)/3
*S* = 1.07	(Δ/σ)_max_ < 0.001
1723 reflections	Δρ_max_ = 0.22 e Å^−^^3^
182 parameters	Δρ_min_ = −0.28 e Å^−^^3^

### Tetraoxa[2]perfluoroarene[2]triazine (I) Special details

**Table:**

Geometry. All esds (except the esd in the dihedral angle between two l.s. planes) are estimated using the full covariance matrix. The cell esds are taken into account individually in the estimation of esds in distances, angles and torsion angles; correlations between esds in cell parameters are only used when they are defined by crystal symmetry. An approximate (isotropic) treatment of cell esds is used for estimating esds involving l.s. planes.

### Tetraoxa[2]perfluoroarene[2]triazine (I) Fractional atomic coordinates and isotropic or equivalent isotropic displacement parameters (Å^2^)

**Table:**

	*x*	*y*	*z*	*U*_iso_*/*U*_eq_	
C1	0.2310 (4)	0.6873 (3)	0.4991 (2)	0.0184 (5)	
C2	0.0922 (4)	0.7559 (3)	0.5196 (2)	0.0182 (5)	
C3	0.1420 (4)	0.8726 (3)	0.6386 (2)	0.0183 (5)	
C4	0.3323 (4)	0.9251 (3)	0.7362 (2)	0.0181 (5)	
C5	0.4723 (4)	0.8573 (3)	0.7158 (2)	0.0180 (5)	
C6	0.4211 (4)	0.7375 (3)	0.5989 (2)	0.0184 (5)	
C7	0.4728 (4)	0.7988 (3)	0.1483 (2)	0.0172 (5)	
C8	0.2802 (4)	0.5391 (3)	0.0409 (2)	0.0174 (5)	
C9	0.0875 (4)	0.2548 (3)	−0.0668 (3)	0.0210 (6)	
H9A	0.159037	0.207379	0.008882	0.032*	
H9B	0.050618	0.188273	−0.162360	0.032*	
H9C	−0.040371	0.250658	−0.040013	0.032*	
C10	0.2633 (4)	0.6098 (3)	0.2654 (2)	0.0174 (5)	
F1	−0.0935 (2)	0.70620 (17)	0.42333 (14)	0.0232 (4)	
F2	0.0058 (2)	0.93768 (17)	0.65671 (14)	0.0237 (4)	
F3	0.6620 (2)	0.91391 (17)	0.80696 (14)	0.0226 (3)	
F4	0.5565 (2)	0.67242 (18)	0.57993 (15)	0.0256 (4)	
N1	0.4026 (3)	0.7671 (2)	0.2726 (2)	0.0166 (4)	
N2	0.4183 (3)	0.6951 (2)	0.0277 (2)	0.0176 (5)	
N3	0.1943 (3)	0.4860 (2)	0.1569 (2)	0.0181 (5)	
O1	0.3814 (2)	1.0421 (2)	0.85763 (16)	0.0197 (4)	
O2	0.2255 (2)	0.4297 (2)	−0.07699 (16)	0.0199 (4)	
O3	0.1720 (2)	0.5621 (2)	0.38295 (17)	0.0211 (4)	

### Tetraoxa[2]perfluoroarene[2]triazine (I) Atomic displacement parameters (Å^2^)

**Table:**

	*U*^11^	*U*^22^	*U*^33^	*U*^12^	*U*^13^	*U*^23^
C1	0.0221 (14)	0.0125 (12)	0.0173 (12)	0.0017 (11)	0.0093 (10)	0.0010 (10)
C2	0.0193 (13)	0.0153 (12)	0.0159 (12)	0.0019 (11)	0.0063 (10)	0.0045 (10)
C3	0.0187 (13)	0.0180 (13)	0.0215 (13)	0.0081 (11)	0.0105 (10)	0.0047 (10)
C4	0.0212 (14)	0.0171 (13)	0.0139 (12)	0.0047 (11)	0.0066 (10)	0.0008 (10)
C5	0.0193 (13)	0.0173 (13)	0.0158 (12)	0.0053 (11)	0.0048 (10)	0.0050 (10)
C6	0.0192 (13)	0.0162 (13)	0.0224 (13)	0.0069 (11)	0.0110 (10)	0.0056 (10)
C7	0.0170 (13)	0.0166 (13)	0.0192 (13)	0.0082 (11)	0.0037 (10)	0.0015 (10)
C8	0.0181 (13)	0.0209 (13)	0.0150 (12)	0.0114 (11)	0.0004 (10)	−0.0017 (10)
C9	0.0228 (13)	0.0165 (13)	0.0214 (13)	0.0068 (11)	0.0027 (10)	−0.0027 (10)
C10	0.0184 (13)	0.0204 (14)	0.0156 (12)	0.0094 (12)	0.0057 (10)	0.0019 (10)
F1	0.0210 (8)	0.0241 (8)	0.0197 (7)	0.0062 (7)	0.0008 (6)	−0.0013 (6)
F2	0.0236 (8)	0.0250 (8)	0.0256 (7)	0.0126 (7)	0.0066 (6)	−0.0017 (6)
F3	0.0209 (8)	0.0252 (8)	0.0199 (7)	0.0089 (7)	0.0023 (6)	0.0002 (6)
F4	0.0264 (8)	0.0252 (8)	0.0298 (8)	0.0140 (7)	0.0097 (6)	−0.0012 (6)
N1	0.0181 (11)	0.0158 (11)	0.0160 (10)	0.0055 (9)	0.0069 (8)	0.0022 (8)
N2	0.0197 (11)	0.0179 (11)	0.0159 (10)	0.0083 (9)	0.0046 (8)	−0.0012 (8)
N3	0.0200 (11)	0.0171 (11)	0.0169 (10)	0.0077 (9)	0.0038 (8)	−0.0016 (8)
O1	0.0240 (9)	0.0178 (9)	0.0148 (8)	0.0046 (8)	0.0078 (7)	−0.0023 (7)
O2	0.0236 (10)	0.0175 (9)	0.0171 (8)	0.0074 (8)	0.0038 (7)	−0.0042 (7)
O3	0.0255 (10)	0.0156 (9)	0.0175 (8)	0.0022 (8)	0.0087 (7)	−0.0025 (7)

### Tetraoxa[2]perfluoroarene[2]triazine (I) Geometric parameters (Å, º)

**Table:**

C1—C6	1.382 (3)	C6—F4	1.338 (2)
C1—C2	1.386 (3)	C7—N2	1.315 (3)
C1—O3	1.392 (3)	C7—N1	1.334 (3)
C2—F1	1.345 (3)	C7—O1^i^	1.360 (3)
C2—C3	1.376 (3)	C8—O2	1.324 (2)
C3—F2	1.342 (2)	C8—N2	1.337 (3)
C3—C4	1.372 (3)	C8—N3	1.338 (3)
C4—C5	1.389 (3)	C9—O2	1.451 (3)
C4—O1	1.396 (2)	C10—N1	1.313 (3)
C5—F3	1.340 (3)	C10—N3	1.325 (3)
C5—C6	1.377 (3)	C10—O3	1.364 (3)
			
C6—C1—C2	119.53 (19)	C5—C6—C1	119.8 (2)
C6—C1—O3	120.98 (19)	N2—C7—N1	128.3 (2)
C2—C1—O3	119.4 (2)	N2—C7—O1^i^	114.5 (2)
F1—C2—C3	119.9 (2)	N1—C7—O1^i^	117.21 (19)
F1—C2—C1	119.63 (19)	O2—C8—N2	114.0 (2)
C3—C2—C1	120.5 (2)	O2—C8—N3	118.8 (2)
F2—C3—C4	120.23 (19)	N2—C8—N3	127.23 (19)
F2—C3—C2	119.7 (2)	N1—C10—N3	129.0 (2)
C4—C3—C2	120.1 (2)	N1—C10—O3	117.82 (19)
C3—C4—C5	119.6 (2)	N3—C10—O3	113.2 (2)
C3—C4—O1	119.94 (19)	C10—N1—C7	111.66 (19)
C5—C4—O1	120.39 (19)	C7—N2—C8	112.2 (2)
F3—C5—C6	119.70 (19)	C10—N3—C8	111.6 (2)
F3—C5—C4	119.79 (19)	C7^i^—O1—C4	116.00 (18)
C6—C5—C4	120.4 (2)	C8—O2—C9	117.07 (18)
F4—C6—C5	120.2 (2)	C10—O3—C1	116.55 (18)
F4—C6—C1	120.02 (19)		
			
C6—C1—C2—F1	−179.5 (2)	C2—C1—C6—C5	−1.2 (4)
O3—C1—C2—F1	−3.0 (3)	O3—C1—C6—C5	−177.7 (2)
C6—C1—C2—C3	−0.3 (4)	N3—C10—N1—C7	2.1 (3)
O3—C1—C2—C3	176.2 (2)	O3—C10—N1—C7	−177.36 (18)
F1—C2—C3—F2	−0.8 (3)	N2—C7—N1—C10	0.6 (3)
C1—C2—C3—F2	180.0 (2)	O1^i^—C7—N1—C10	−179.86 (19)
F1—C2—C3—C4	−179.3 (2)	N1—C7—N2—C8	−2.5 (3)
C1—C2—C3—C4	1.5 (4)	O1^i^—C7—N2—C8	177.90 (18)
F2—C3—C4—C5	−179.7 (2)	O2—C8—N2—C7	−178.34 (18)
C2—C3—C4—C5	−1.2 (4)	N3—C8—N2—C7	2.4 (3)
F2—C3—C4—O1	2.2 (3)	N1—C10—N3—C8	−2.2 (3)
C2—C3—C4—O1	−179.3 (2)	O3—C10—N3—C8	177.27 (18)
C3—C4—C5—F3	176.9 (2)	O2—C8—N3—C10	−179.58 (19)
O1—C4—C5—F3	−5.0 (3)	N2—C8—N3—C10	−0.3 (3)
C3—C4—C5—C6	−0.3 (4)	C3—C4—O1—C7^i^	−108.0 (3)
O1—C4—C5—C6	177.8 (2)	C5—C4—O1—C7^i^	74.0 (3)
F3—C5—C6—F4	2.9 (3)	N2—C8—O2—C9	175.75 (18)
C4—C5—C6—F4	−179.9 (2)	N3—C8—O2—C9	−4.9 (3)
F3—C5—C6—C1	−175.7 (2)	N1—C10—O3—C1	1.8 (3)
C4—C5—C6—C1	1.5 (4)	N3—C10—O3—C1	−177.71 (18)
C2—C1—C6—F4	−179.8 (2)	C6—C1—O3—C10	−77.2 (3)
O3—C1—C6—F4	3.7 (4)	C2—C1—O3—C10	106.4 (2)

Symmetry code: (i) −*x*+1, −*y*+2, −*z*+1.

### (II) Crystal data

**Table:**

C_20_H_6_F_8_N_6_O_6_	*Z* = 2
*M**_r_* = 578.31	*F*(000) = 576
Triclinic, *P*1	*D*_x_ = 1.770 Mg m^−^^3^
*a* = 7.1729 (10) Å	Mo *K*α radiation, λ = 0.71073 Å
*b* = 9.7282 (12) Å	Cell parameters from 3736 reflections
*c* = 16.307 (2) Å	θ = 2.2–25.3°
α = 104.420 (4)°	µ = 0.18 mm^−^^1^
β = 91.293 (5)°	*T* = 100 K
γ = 99.274 (4)°	Prismatic, colourless
*V* = 1085.3 (3) Å^3^	0.25 × 0.19 × 0.09 mm

### (II) Data collection

**Table:**

Bruker D8 Goniometer diffractometer	2824 reflections with *I* > 2σ(*I*)
Detector resolution: 7.3910 pixels mm^-1^	*R*_int_ = 0.053
φ and ω scans	θ_max_ = 25.0°, θ_min_ = 2.6°
Absorption correction: multi-scan (SADABS; Krause et al., 2015)	*h* = −8→7
*T*_min_ = 0.74, *T*_max_ = 0.98	*k* = −11→11
10388 measured reflections	*l* = −19→17
3800 independent reflections	

### (II) Refinement

**Table:**

Refinement on *F*^2^	Hydrogen site location: inferred from neighbouring sites
Least-squares matrix: full	H-atom parameters constrained
*R*[*F*^2^ > 2σ(*F*^2^)] = 0.047	*w* = 1/[σ^2^(*F*_o_^2^) + (0.0502*P*)^2^ + 0.5197*P*] where *P* = (*F*_o_^2^ + 2*F*_c_^2^)/3
*wR*(*F*^2^) = 0.130	(Δ/σ)_max_ < 0.001
*S* = 1.03	Δρ_max_ = 0.26 e Å^−^^3^
3800 reflections	Δρ_min_ = −0.24 e Å^−^^3^
364 parameters	Extinction correction: *SHELXL2019/1* (Sheldrick, 2015b), Fc^*^=kFc[1+0.001xFc^2^λ^3^/sin(2θ)]^-1/4^
0 restraints	Extinction coefficient: 0.011 (2)

### (II) Special details

**Table:**

Geometry. All esds (except the esd in the dihedral angle between two l.s. planes) are estimated using the full covariance matrix. The cell esds are taken into account individually in the estimation of esds in distances, angles and torsion angles; correlations between esds in cell parameters are only used when they are defined by crystal symmetry. An approximate (isotropic) treatment of cell esds is used for estimating esds involving l.s. planes.

### (II) Fractional atomic coordinates and isotropic or equivalent isotropic displacement parameters (Å^2^)

**Table:**

	*x*	*y*	*z*	*U*_iso_*/*U*_eq_	
C1	−0.0322 (4)	0.7241 (3)	0.43944 (16)	0.0287 (6)	
C2	0.1465 (4)	0.7223 (3)	0.47154 (18)	0.0330 (6)	
C3	0.2460 (4)	0.6168 (3)	0.43252 (18)	0.0333 (6)	
C4	0.1702 (4)	0.5124 (3)	0.36165 (17)	0.0304 (6)	
C5	−0.0089 (4)	0.5143 (3)	0.32978 (16)	0.0328 (6)	
C6	−0.1094 (4)	0.6188 (3)	0.36824 (17)	0.0321 (6)	
C7	0.2848 (4)	0.2983 (3)	0.35344 (16)	0.0300 (6)	
C8	0.3974 (4)	0.1003 (3)	0.34991 (16)	0.0274 (6)	
C9	0.5368 (4)	−0.1050 (3)	0.34988 (18)	0.0373 (7)	
H9A	0.579009	−0.063645	0.409822	0.056*	
H9B	0.634031	−0.155031	0.320517	0.056*	
H9C	0.418553	−0.173587	0.345406	0.056*	
C10	0.2161 (4)	0.1821 (3)	0.45146 (16)	0.0258 (6)	
C11	0.6456 (4)	0.3086 (3)	0.06544 (16)	0.0311 (6)	
C12	0.4606 (4)	0.2599 (3)	0.07821 (18)	0.0374 (7)	
C13	0.3495 (4)	0.3539 (3)	0.12138 (18)	0.0364 (7)	
C14	0.4226 (4)	0.4977 (3)	0.15294 (16)	0.0305 (6)	
C15	0.6086 (4)	0.5459 (3)	0.14003 (17)	0.0316 (6)	
C16	0.7179 (4)	0.4530 (3)	0.09641 (17)	0.0315 (6)	
C17	0.2471 (4)	0.6855 (3)	0.16635 (17)	0.0307 (6)	
C18	0.0764 (4)	0.8572 (3)	0.17797 (18)	0.0345 (7)	
C19	−0.1149 (5)	1.0382 (3)	0.1883 (3)	0.0610 (10)	
H19A	−0.017221	1.101278	0.167413	0.092*	
H19B	−0.183562	1.096947	0.230976	0.092*	
H19C	−0.203541	0.980919	0.140994	0.092*	
C20	0.2116 (4)	0.7792 (3)	0.05912 (18)	0.0322 (6)	
F1	0.2200 (2)	0.81948 (18)	0.54274 (11)	0.0482 (5)	
F2	0.4177 (2)	0.61428 (19)	0.46579 (12)	0.0499 (5)	
F3	−0.0880 (3)	0.41263 (19)	0.26064 (10)	0.0501 (5)	
F4	−0.2852 (2)	0.61680 (19)	0.33718 (10)	0.0475 (5)	
F5	0.3847 (3)	0.12112 (17)	0.04626 (13)	0.0623 (6)	
F6	0.1679 (2)	0.30503 (18)	0.13105 (13)	0.0541 (5)	
F7	0.6813 (2)	0.68678 (17)	0.16853 (11)	0.0461 (5)	
F8	0.8964 (2)	0.50253 (18)	0.08165 (11)	0.0465 (5)	
N1	0.1923 (3)	0.2948 (2)	0.42308 (13)	0.0265 (5)	
N2	0.3882 (3)	0.2074 (2)	0.31248 (14)	0.0321 (5)	
N3	0.3139 (3)	0.0802 (2)	0.41949 (13)	0.0280 (5)	
N4	0.2903 (3)	0.6847 (2)	0.08793 (13)	0.0283 (5)	
N5	0.1441 (3)	0.7670 (2)	0.21600 (14)	0.0344 (6)	
N6	0.1023 (3)	0.8675 (2)	0.09876 (16)	0.0383 (6)	
O1	0.2731 (3)	0.4108 (2)	0.31921 (12)	0.0364 (5)	
O2	0.5057 (3)	0.00888 (19)	0.31135 (12)	0.0347 (5)	
O3	0.1319 (3)	0.16776 (19)	0.52332 (11)	0.0324 (5)	
O4	0.3142 (3)	0.58860 (19)	0.20191 (11)	0.0342 (5)	
O5	−0.0266 (3)	0.9427 (2)	0.22636 (14)	0.0444 (5)	
O6	0.2438 (3)	0.7868 (2)	−0.02171 (12)	0.0382 (5)	

### (II) Atomic displacement parameters (Å^2^)

**Table:**

	*U*^11^	*U*^22^	*U*^33^	*U*^12^	*U*^13^	*U*^23^
C1	0.0333 (16)	0.0314 (14)	0.0310 (14)	0.0157 (11)	0.0154 (12)	0.0181 (12)
C2	0.0325 (16)	0.0320 (14)	0.0357 (16)	0.0068 (11)	0.0065 (12)	0.0098 (13)
C3	0.0233 (15)	0.0412 (16)	0.0426 (17)	0.0114 (12)	0.0075 (12)	0.0198 (14)
C4	0.0375 (17)	0.0327 (14)	0.0318 (15)	0.0189 (12)	0.0168 (12)	0.0189 (12)
C5	0.0440 (18)	0.0348 (15)	0.0230 (14)	0.0154 (12)	0.0061 (12)	0.0080 (12)
C6	0.0294 (16)	0.0426 (16)	0.0313 (15)	0.0162 (12)	0.0041 (12)	0.0156 (13)
C7	0.0310 (16)	0.0351 (15)	0.0296 (14)	0.0147 (12)	0.0053 (12)	0.0131 (12)
C8	0.0257 (15)	0.0291 (14)	0.0273 (14)	0.0111 (11)	−0.0001 (11)	0.0035 (11)
C9	0.0459 (19)	0.0319 (15)	0.0392 (16)	0.0210 (13)	0.0081 (14)	0.0089 (13)
C10	0.0238 (14)	0.0305 (14)	0.0254 (13)	0.0080 (10)	0.0030 (11)	0.0094 (11)
C11	0.0361 (17)	0.0369 (15)	0.0253 (14)	0.0139 (12)	0.0089 (12)	0.0117 (12)
C12	0.0448 (19)	0.0270 (14)	0.0401 (16)	0.0047 (12)	0.0131 (14)	0.0082 (13)
C13	0.0297 (16)	0.0408 (16)	0.0406 (16)	0.0026 (12)	0.0152 (13)	0.0149 (13)
C14	0.0335 (16)	0.0378 (15)	0.0240 (13)	0.0108 (12)	0.0083 (12)	0.0114 (12)
C15	0.0325 (16)	0.0342 (15)	0.0282 (14)	0.0041 (12)	0.0037 (12)	0.0093 (12)
C16	0.0224 (15)	0.0432 (16)	0.0313 (14)	0.0047 (12)	0.0051 (11)	0.0144 (12)
C17	0.0268 (15)	0.0313 (14)	0.0345 (15)	0.0021 (11)	0.0056 (12)	0.0110 (12)
C18	0.0300 (16)	0.0286 (14)	0.0405 (17)	0.0020 (11)	0.0133 (13)	0.0017 (13)
C19	0.064 (2)	0.0414 (18)	0.090 (3)	0.0282 (17)	0.042 (2)	0.0258 (19)
C20	0.0298 (16)	0.0311 (14)	0.0369 (16)	0.0055 (11)	0.0085 (12)	0.0100 (12)
F1	0.0395 (11)	0.0452 (10)	0.0525 (11)	0.0088 (8)	0.0003 (8)	−0.0022 (8)
F2	0.0295 (10)	0.0610 (11)	0.0607 (12)	0.0191 (8)	0.0009 (8)	0.0115 (9)
F3	0.0550 (12)	0.0573 (11)	0.0366 (10)	0.0278 (9)	0.0007 (8)	−0.0016 (8)
F4	0.0413 (11)	0.0630 (11)	0.0412 (10)	0.0298 (8)	−0.0030 (8)	0.0059 (8)
F5	0.0613 (13)	0.0313 (9)	0.0865 (15)	0.0000 (8)	0.0327 (11)	0.0035 (9)
F6	0.0368 (11)	0.0478 (10)	0.0715 (13)	−0.0016 (8)	0.0238 (9)	0.0078 (9)
F7	0.0416 (11)	0.0374 (9)	0.0515 (11)	−0.0015 (7)	0.0097 (8)	0.0015 (8)
F8	0.0291 (10)	0.0523 (10)	0.0527 (11)	0.0030 (7)	0.0112 (8)	0.0055 (8)
N1	0.0250 (12)	0.0324 (12)	0.0281 (12)	0.0119 (9)	0.0078 (9)	0.0138 (10)
N2	0.0359 (14)	0.0361 (13)	0.0314 (12)	0.0192 (10)	0.0106 (10)	0.0127 (10)
N3	0.0294 (13)	0.0298 (12)	0.0276 (12)	0.0129 (9)	0.0062 (9)	0.0072 (9)
N4	0.0261 (12)	0.0329 (12)	0.0277 (12)	0.0058 (9)	0.0066 (9)	0.0104 (10)
N5	0.0341 (14)	0.0331 (12)	0.0349 (13)	0.0063 (10)	0.0116 (11)	0.0055 (10)
N6	0.0361 (15)	0.0337 (13)	0.0495 (15)	0.0115 (10)	0.0169 (12)	0.0143 (11)
O1	0.0456 (13)	0.0429 (11)	0.0356 (11)	0.0283 (9)	0.0208 (9)	0.0227 (9)
O2	0.0391 (12)	0.0358 (10)	0.0352 (10)	0.0214 (8)	0.0112 (9)	0.0101 (8)
O3	0.0393 (12)	0.0346 (10)	0.0320 (10)	0.0198 (8)	0.0145 (8)	0.0151 (8)
O4	0.0383 (12)	0.0391 (11)	0.0298 (10)	0.0138 (8)	0.0127 (8)	0.0117 (8)
O5	0.0444 (13)	0.0343 (11)	0.0563 (13)	0.0138 (9)	0.0244 (10)	0.0084 (10)
O6	0.0424 (13)	0.0426 (11)	0.0393 (11)	0.0204 (9)	0.0160 (9)	0.0187 (9)

### (II) Geometric parameters (Å, º)

**Table:**

C1—C2	1.378 (4)	C11—C12	1.374 (4)
C1—C6	1.378 (4)	C11—C16	1.378 (4)
C1—O3^i^	1.390 (3)	C11—O6^ii^	1.390 (3)
C2—F1	1.336 (3)	C12—F5	1.338 (3)
C2—C3	1.378 (4)	C12—C13	1.381 (4)
C3—F2	1.341 (3)	C13—F6	1.340 (3)
C3—C4	1.368 (4)	C13—C14	1.374 (4)
C4—C5	1.379 (4)	C14—C15	1.380 (4)
C4—O1	1.385 (3)	C14—O4	1.386 (3)
C5—F3	1.344 (3)	C15—F7	1.344 (3)
C5—C6	1.374 (4)	C15—C16	1.368 (4)
C6—F4	1.343 (3)	C16—F8	1.343 (3)
C7—N2	1.311 (3)	C17—N5	1.313 (3)
C7—N1	1.333 (3)	C17—N4	1.321 (3)
C7—O1	1.360 (3)	C17—O4	1.365 (3)
C8—O2	1.325 (3)	C18—O5	1.326 (3)
C8—N3	1.337 (3)	C18—N6	1.335 (4)
C8—N2	1.341 (3)	C18—N5	1.336 (4)
C9—O2	1.447 (3)	C19—O5	1.452 (4)
C10—N3	1.316 (3)	C20—N6	1.317 (3)
C10—N1	1.324 (3)	C20—N4	1.325 (3)
C10—O3	1.358 (3)	C20—O6	1.361 (3)
			
C2—C1—C6	119.0 (2)	F6—C13—C14	119.7 (2)
C2—C1—O3^i^	120.8 (2)	F6—C13—C12	119.7 (2)
C6—C1—O3^i^	120.2 (2)	C14—C13—C12	120.6 (3)
F1—C2—C3	120.0 (2)	C13—C14—C15	118.6 (2)
F1—C2—C1	119.8 (2)	C13—C14—O4	119.9 (2)
C3—C2—C1	120.2 (3)	C15—C14—O4	121.4 (2)
F2—C3—C4	119.5 (2)	F7—C15—C16	119.7 (2)
F2—C3—C2	119.5 (3)	F7—C15—C14	119.5 (2)
C4—C3—C2	121.0 (3)	C16—C15—C14	120.8 (3)
C3—C4—C5	118.7 (2)	F8—C16—C15	120.1 (2)
C3—C4—O1	121.4 (2)	F8—C16—C11	119.1 (2)
C5—C4—O1	119.8 (2)	C15—C16—C11	120.8 (3)
F3—C5—C6	119.0 (2)	N5—C17—N4	128.7 (3)
F3—C5—C4	120.2 (2)	N5—C17—O4	114.0 (2)
C6—C5—C4	120.8 (2)	N4—C17—O4	117.3 (2)
F4—C6—C5	119.8 (2)	O5—C18—N6	119.2 (3)
F4—C6—C1	119.9 (2)	O5—C18—N5	114.1 (2)
C5—C6—C1	120.3 (2)	N6—C18—N5	126.7 (2)
N2—C7—N1	128.8 (2)	N6—C20—N4	128.0 (3)
N2—C7—O1	114.3 (2)	N6—C20—O6	114.7 (2)
N1—C7—O1	116.9 (2)	N4—C20—O6	117.3 (2)
O2—C8—N3	119.5 (2)	C10—N1—C7	111.6 (2)
O2—C8—N2	113.1 (2)	C7—N2—C8	111.6 (2)
N3—C8—N2	127.4 (2)	C10—N3—C8	112.1 (2)
N3—C10—N1	128.4 (2)	C17—N4—C20	111.8 (2)
N3—C10—O3	114.1 (2)	C17—N5—C18	112.2 (2)
N1—C10—O3	117.4 (2)	C20—N6—C18	112.5 (2)
C12—C11—C16	118.7 (2)	C7—O1—C4	116.5 (2)
C12—C11—O6^ii^	120.2 (2)	C8—O2—C9	117.8 (2)
C16—C11—O6^ii^	121.1 (2)	C10—O3—C1^i^	115.88 (19)
F5—C12—C11	120.0 (2)	C17—O4—C14	117.4 (2)
F5—C12—C13	119.4 (3)	C18—O5—C19	117.7 (2)
C11—C12—C13	120.6 (2)	C20—O6—C11^ii^	115.5 (2)
			
C6—C1—C2—F1	−176.9 (2)	C12—C11—C16—F8	−177.7 (2)
O3^i^—C1—C2—F1	4.7 (4)	O6^ii^—C11—C16—F8	2.0 (4)
C6—C1—C2—C3	0.2 (4)	C12—C11—C16—C15	0.9 (4)
O3^i^—C1—C2—C3	−178.1 (2)	O6^ii^—C11—C16—C15	−179.4 (2)
F1—C2—C3—F2	−1.2 (4)	N3—C10—N1—C7	−0.5 (4)
C1—C2—C3—F2	−178.3 (2)	O3—C10—N1—C7	178.7 (2)
F1—C2—C3—C4	177.2 (2)	N2—C7—N1—C10	0.7 (4)
C1—C2—C3—C4	0.0 (4)	O1—C7—N1—C10	−179.0 (2)
F2—C3—C4—C5	178.1 (2)	N1—C7—N2—C8	−0.8 (4)
C2—C3—C4—C5	−0.2 (4)	O1—C7—N2—C8	178.9 (2)
F2—C3—C4—O1	−5.4 (4)	O2—C8—N2—C7	−178.1 (2)
C2—C3—C4—O1	176.3 (2)	N3—C8—N2—C7	0.8 (4)
C3—C4—C5—F3	−179.5 (2)	N1—C10—N3—C8	0.6 (4)
O1—C4—C5—F3	4.0 (4)	O3—C10—N3—C8	−178.7 (2)
C3—C4—C5—C6	0.1 (4)	O2—C8—N3—C10	178.2 (2)
O1—C4—C5—C6	−176.4 (2)	N2—C8—N3—C10	−0.7 (4)
F3—C5—C6—F4	0.9 (4)	N5—C17—N4—C20	−1.4 (4)
C4—C5—C6—F4	−178.6 (2)	O4—C17—N4—C20	177.6 (2)
F3—C5—C6—C1	179.8 (2)	N6—C20—N4—C17	0.0 (4)
C4—C5—C6—C1	0.2 (4)	O6—C20—N4—C17	−179.1 (2)
C2—C1—C6—F4	178.5 (2)	N4—C17—N5—C18	0.9 (4)
O3^i^—C1—C6—F4	−3.2 (4)	O4—C17—N5—C18	−178.1 (2)
C2—C1—C6—C5	−0.4 (4)	O5—C18—N5—C17	−179.2 (2)
O3^i^—C1—C6—C5	178.0 (2)	N6—C18—N5—C17	1.0 (4)
C16—C11—C12—F5	177.6 (3)	N4—C20—N6—C18	1.5 (4)
O6^ii^—C11—C12—F5	−2.1 (4)	O6—C20—N6—C18	−179.3 (2)
C16—C11—C12—C13	−0.1 (4)	O5—C18—N6—C20	178.2 (2)
O6^ii^—C11—C12—C13	−179.8 (3)	N5—C18—N6—C20	−2.1 (4)
F5—C12—C13—F6	0.3 (4)	N2—C7—O1—C4	−178.1 (2)
C11—C12—C13—F6	178.1 (3)	N1—C7—O1—C4	1.6 (4)
F5—C12—C13—C14	−178.3 (3)	C3—C4—O1—C7	79.5 (3)
C11—C12—C13—C14	−0.5 (4)	C5—C4—O1—C7	−104.1 (3)
F6—C13—C14—C15	−178.2 (2)	N3—C8—O2—C9	−3.4 (4)
C12—C13—C14—C15	0.4 (4)	N2—C8—O2—C9	175.6 (2)
F6—C13—C14—O4	6.5 (4)	N3—C10—O3—C1^i^	178.0 (2)
C12—C13—C14—O4	−174.9 (3)	N1—C10—O3—C1^i^	−1.3 (3)
C13—C14—C15—F7	178.3 (2)	N5—C17—O4—C14	179.3 (2)
O4—C14—C15—F7	−6.5 (4)	N4—C17—O4—C14	0.2 (3)
C13—C14—C15—C16	0.3 (4)	C13—C14—O4—C17	−108.6 (3)
O4—C14—C15—C16	175.5 (2)	C15—C14—O4—C17	76.3 (3)
F7—C15—C16—F8	−0.5 (4)	N6—C18—O5—C19	3.3 (4)
C14—C15—C16—F8	177.5 (2)	N5—C18—O5—C19	−176.5 (3)
F7—C15—C16—C11	−179.0 (2)	N6—C20—O6—C11^ii^	−178.2 (2)
C14—C15—C16—C11	−1.0 (4)	N4—C20—O6—C11^ii^	1.0 (3)

Symmetry codes: (i) −*x*, −*y*+1, −*z*+1; (ii) −*x*+1, −*y*+1, −*z*.
